# A Machine Learning Approach to Predict the Outcome of Urinary Calculi Treatment Using Shock Wave Lithotripsy: Model Development and Validation Study

**DOI:** 10.2196/33357

**Published:** 2022-03-16

**Authors:** Reihaneh Moghisi, Christo El Morr, Kenneth T Pace, Mohammad Hajiha, Jimmy Huang

**Affiliations:** 1 School of Information Technology York University Toronto, ON Canada; 2 School of Health Policy and Management York University Toronto, ON Canada; 3 Division of Urology St Michael's Hospital Toronto, ON Canada; 4 Department of Surgery University of Toronto Toronto, ON Canada; 5 Department of Urology Loma Linda University Health Loma Linda, CA United States

**Keywords:** lithotripsy, urolithiasis, machine learning, treatment outcome, ensemble learning, AdaBoost, renal stones, kidney disease

## Abstract

**Background:**

Shock wave lithotripsy (SWL), ureteroscopy, and percutaneous nephrolithotomy are established treatments for renal stones. Historically, SWL has been a predominant and commonly used procedure for treating upper tract renal stones smaller than 20 mm in diameter due to its noninvasive nature. However, the reported failure rate of SWL after one treatment session ranges from 30% to 89%. The failure rate can be reduced by identifying candidates likely to benefit from SWL and manage patients who are likely to fail SWL with other treatment modalities. This would enhance and optimize treatment results for SWL candidates.

**Objective:**

We proposed to develop a machine learning model that can predict SWL outcomes to assist practitioners in the decision-making process when considering patients for stone treatment.

**Methods:**

A data set including 58,349 SWL procedures performed during 31,569 patient visits for SWL to a single hospital between 1990 and 2016 was used to construct and validate the predictive model. The AdaBoost algorithm was applied to a data set with 17 predictive attributes related to patient demographics and stone characteristics, with success or failure as an outcome. The AdaBoost algorithm was also applied to a training data set. The generated model’s performance was compared to that of 5 other machine learning algorithms, namely C4.5 decision tree, naïve Bayes, Bayesian network, K-nearest neighbors, and multilayer perceptron.

**Results:**

The developed model was validated with a testing data set and performed significantly better than the models generated by the other 5 predictive algorithms. The sensitivity and specificity of the model were 0.875 and 0.653, respectively, while its positive predictive value was 0.7159 and negative predictive value was 0.839. The C-statistics of the receiver operating characteristic (ROC) analysis was 0.843, which reflects an excellent test.

**Conclusions:**

We have developed a rigorous machine learning model to assist physicians and decision-makers to choose patients with renal stones who are most likely to have successful SWL treatment based on their demographics and stone characteristics. The proposed machine learning model can assist physicians and decision-makers in planning for SWL treatment and allow for more effective use of limited health care resources and improve patient prognoses.

## Introduction

Urinary stone disease, also known as urolithiasis, is a disease that occurs when a solid particle of minerals and salts is formed inside the urinary tract. A recent systematic review suggests an increasing prevalence of urolithiasis in North America over the past 3 decades [1]. In Canada, urinary stone disease is prevalent with a lifetime risk of 10% among both men and women, whereas there is a 75% chance of recurrence in 20 years after initial treatment [2].

Historically, shock wave lithotripsy (SWL) has been the most used procedure for treating upper tract urolithiasis and stones smaller than 20 mm in diameter due to its noninvasive nature, lower cost, fewer side effects, and faster recovery [3,4].

In Ontario, Canada, SWL is a regionalized and limited resource. St. Michael’s Hospital in downtown Toronto is one of the only 3 centers in the province offering this service. Wait time to access SWL treatment in Canada ranges from 1 day to 1 year, with a mean wait time of 8.4 weeks in Ottawa and 8 weeks in Toronto [5]. Considering the intolerability of the pain associated with stone disease and long wait times, some patients opt for more invasive therapies such as ureteroscopy to gain access to faster treatment.

While SWL is the predominant treatment, the reported failure rate of SWL after the first session ranges from 30% to 89% [6-8]. The failure rate can be reduced significantly by identifying the candidates who are most likely to benefit from SWL, which would optimize treatment results for SWL candidates and allow for the most effective use of limited medical resources.

To identify the predictive factors of SWL outcome, several studies have focused on statistical analyses of patient characteristics using bivariate and/or multivariate analysis [4,9-11]. The advantage and strength of machine learning is its ability to synthesize complex combinations of various attributes [12,13]. Our objective for this study was to construct a robust machine learning model that can predict SWL results to assist practitioners in their decision-making.

## Methods

### Ethics Approval

This study received ethics approval from the Office of Research Ethics at York University (certificate number STU 2019-139) and St. Michael Research Ethics Board (approval number 16-167).

### Data Set

We assessed a data set of patients aged ≥18 years receiving SWL treatment at St. Michael’s Hospital between 1998 and 2016. The data set comprised the records of 37,013 patients.

We excluded the data of patients with special conditions (eg, staghorn calculi, horseshoe kidney, caliceal diverticula, duplex collecting systems, solitary kidneys, musculoskeletal deformities) and stones larger than 25 mm in diameter. The remaining data set consisted of 57,485 SWL procedures that were performed on 31,569 patients during this period, which were used as a training data set to build the model. Several factors can impact SWL treatment outcome, including stone location and age; the choice of the attributes was guided by input from clinical experts and a literature review [4,10,11,14]. We retained 17 attributes that were most relevant to SWL success and were available in our database (Table 1).

**Table 1:** Training set attributes and corresponding values.

Attribute	Value
Kidney side	Left or right
Electrode used	Integer (1 to 3)
Stone treatment number	Integer
Number of shocks	Integer
Stone locations	Lower calyx, lower ureter, middle calyx, middle ureter, pelvis, upper calyx, upper ureter, ureterovesical junction, renal pelvis
Area of stone	Integer (mm^2^)
Gender	Female or male
BMI	Real number (kg/m^2^)
Age	18-95
Number of stones	Integer
Family history	True or false
Asymptomatic	True or false
Stent insertion	True or false
Shock frequency	120, 90, 60
Antibiotic	True or false
Shock maximum voltage	Integer
Lithotripter models	Dornier MFL 5000, Philips LithoTron, Storz Modulith SLX-F2
Outcome	Success or failure

### Defining Success and Failure of SWL on the Training Data Set

The failure or success of SWL in the training data set was based on whether there was a retreatment plan for the same patient and same stone within 90 days after initial treatment or not. The effectiveness of the lithotripter machine was measured by success rates on the training set.

### Ensemble Learning Technique

To predict the treatment outcome for SWL candidates, we used the AdaBoost algorithm based on the ensemble learning method, a machine learning technique that combines several base classifiers in various formats to produce a more robust and optimal classification model. Compared to other conventional machine learning algorithms, ensemble learning techniques are more stable, faster, simpler, and easier to program [15-19].

AdaBoost combines multiple weak classifiers that are sequentially applied to the data set. In each iteration, after the weak classifier is called, misclassified item sets are detected and given higher weight to increase the emphasis of the weak classifier on them in the next round. The final classification model is then generated as a linear combination of these weak classifiers with their assigned weights as their coefficient [19]. We used 10-fold cross-validation for AdaBoost.

### Performance Evaluation

To compare AdaBoost’s performance to that of other classifiers, we used 5 classification algorithms to predict SWL failure (retreatment required <3 months), namely C4.5, naïve Bayes, Bayesian network, K-nearest neighbors, and multilayer perceptron, and used *t* tests to perform pairwise comparisons of the performance of the AdaBoost algorithm against that of the other 5 classification models. The measurements used to determine the models’ performance were sensitivity, specificity, positive predictive value (PPV), negative predictive value (NPV) [20], accuracy, F1 score [14,21], and Matthews correlation coefficient [22]. Machine learning was performed using WEKA (version 3.9; University of Waikato) [23]. We used 10-fold cross-validation for performance evaluation.

### Generalizability of the Model

Classifiers were assessed for generalizability using the testing data set of 864 patients who had their preoperative and postoperative follow-ups conducted at the same center, and whose SWL procedure success and failure was determined by computed tomography (CT) scan of patients 3 months after the initial therapy. The testing data set was not included in the training set used to build the model. We employed the undersampling technique to resolve the imbalance in data by removing random examples from the majority class. *SpreadSubsample* was the Java class implemented for subsampling the original training set. We matched the ratio of success to failure in the training set to the ratio observed in our testing set, which was 40% to 60%.

## Results

### The AdaBoost Model

A total of 30 iterations were used for the AdaBoost model. Although increasing the number of iterations usually increases the accuracy of the model, we ceased adding more iterations to the model to avoid overfitting.

Research has shown that applying the boosting method to any weak classifier can drastically enhance the accuracy of the classification model [24]. Indeed, the accuracy of applying the base learner (Decision Stump) alone on our data set was 67.8%. However, with the ensemble method, we could boost this accuracy by 9% to 76.38%, which demonstrates the superiority of the boosting method.

### Model Performance

Table 2 shows the comparison of the AdaBoost model against the other 5 classification techniques in terms of 4 different performance measurements. AdaBoost performed significantly better than all 5 other classifiers on all performance measures.

**Table 2:** Performance comparison of AdaBoost against 5 other classifiers.

Measurement	AdaBoostM1	C4.5	Naïve Bayes	Multilayer perceptron	Bayesian network	KNN^a^
Accuracy	77.59	75.26^b^	75.82^b^	69.11^b^	76.49^b^	57.52^b^
MCC^c^	0.53	0.46^b^	0.47^b^	0.34^b^	0.49^b^	0.09^b^
F1 score	0.84	0.82^b^	0.83^b^	0.76^b^	0.83^b^	0.66^b^
Area under ROC^d^	0.80	0.74^b^	0.75^b^	0.74^b^	0.78^b^	0.54^b^

^a^KNN: K-nearest neighbors.

^b^Statistically significant.

^c^MCC: Matthews correlation coefficient.

^d^ROC: receiver operating characteristic.

The sensitivity of the model was 0.875 (ie, 87.5% of all patients with successful SWL treatment were correctly identified by our model). On the other hand, the specificity was 0.6528 (ie, 65.3% of all patients with failed SWL treatment were correctly identified by our model).

Furthermore, the PPV (ie, the probability that subjects with a success prediction truly succeeded in the treatment) was 0.7159. Meanwhile, the NPV (ie, the probability that subjects with a failure prediction have truly failed the treatment) was 0.839.

Finally, we measured the correlation between the attributes and the class; the top 5 contributors detected were the number of stones, the area of the stone, the stone treatment number, the lithotripter machine, and the patient’s age.

## Discussion

### Principal Findings

Our goal was to evaluate the ability of machine learning techniques to assist in effective decision-making for the treatment of urolithiasis with SWL by accurately predicting the SWL results. We have shown that AdaBoost provided superior prediction ability compared to 5 other classification techniques.

The AUC (area under the ROC [receiver operating characteristic] curve or C-statistic) of the ROC analysis for our prediction model was 0.843, which reflects an excellent test (a C-statistic value of 0.8-0.89 indicates an excellent test, 0.7-0.79 indicates a good test, and 0.51-0.69 indicates a poor test) [25].

The model had high sensitivity and medium specificity. Given that we are interested in identifying the patients for whom SWL has a low chance of success to plan for alternative procedures, the NPV of 0.839 demonstrated that the model can predict with high probability if a subject will fail the treatment. Considering how scarce and expensive health care resources are, it is important to allocate those limited resources appropriately [26,27]; our model allows for appropriate allocation by informing physicians about patients who are not likely to benefit from SWL.

Recently, Choo et al [28] developed a decision tree algorithm C 5.0 for the same purpose of predicting treatment outcomes for SWL, including 15 predictive attributes on only 791 patients. Although their model had high accuracy (92.3%), some of its branches included fewer than 10 patients each. Considering that our AdaBoost-based model outperformed the decision tree algorithm in all performance measurements, we can expect it to yield better accuracy if other predictive attributes (ie, skin-to-stone distance, stone Hounsfield unit, creatinine level, stone composition, etc [3,4]) were included in the data set in a future study.

Our results show that the 3 different models of lithotripters did not significantly change the SWL treatment success rate (*P*=.81). This finding suggests that frequently upgrading the technology of SWL machines does not necessarily result in a better outcome, whereas optimizing patient and stone selection is a more important factor in predicting the outcome of the SWL.

### Limitations

A limitation of this study was the lack of follow-up data for some of the patients enrolled. As a result, a treatment’s failure was defined only based on having retreatment of a stone in the same center (St. Michael’s Hospital) within 3 months of the initial SWL. However, to overcome this limitation and test the robustness of our model, we used 864 records that included only patients who had their complete preoperative and postoperative follow-ups conducted at St. Michael’s Hospital. This subset of the data set was not used for training the model. The follow-up data, the stone-free rate, and the success of treatment for these patients were assessed based on the follow-up CT scan administered at St. Michael’s Hospital 3 months after the initial SWL.

Another limitation is that some attributes that have been shown to be predictive of SWL outcome in recent studies, such as stone density, skin-to-stone distance, and stone composition [10], were not available in our database since these data points were not known or collected 20 years ago.

### Conclusion

We built a machine learning model to assist physicians and decision-makers to choose the best treatment option for SWL candidates based on their demographics and stone characteristics, which can result in improved prognoses. The model was generated based on the AdaBoost algorithm.

A pairwise comparison was performed between the AdaBoost classifier and 5 other classification techniques in terms of their accuracy, Matthews correlation coefficient, area under the ROC curve, and root mean squared error. The findings of these comparisons suggest the superiority of AdaBoost compared to those algorithms.

We aim to explore several meaningful research directions in the future. First, we will develop new models and architectures that are more robust and efficient by utilizing deep learning techniques. Second, our proposed ensemble learning approach can be applied to more comprehensive databases for more applications to ascertain the applicability of the model [29-32].

## Abbreviations

AUC: area under the receiver operating characteristic curve
CT: computed tomography
NPV: negative predictive value
PPV: positive predictive value
ROC: receiver operating characteristic
SWL: shock wave lithotripsy

## Data Availability

The data sets generated and/or analyzed in this study are not publicly available due to patient data privacy reasons but are available from the corresponding author upon reasonable request.
